# Autonomic nervous system balance in parturient mares: Spontaneous vs induced delivery

**DOI:** 10.1371/journal.pone.0283116

**Published:** 2023-03-17

**Authors:** Martina Felici, Micaela Sgorbini, Paolo Baragli, Antonio Lanatà, Paola Marmorini, Francesco Camillo

**Affiliations:** 1 Department of Veterinary Sciences, University of Pisa, Pisa, Italy; 2 Department of Agricultural and Food Science (DISTAL), University of Bologna, Bologna, Italy; 3 Veterinary Teaching Hospital “Mario Modenato”, University of Pisa, Pisa, Italy; 4 Department of Information Engineering, University of Florence, Firenze, Italy; 5 Private Practitioner, Pisa, Italy; Universita Politecnica delle Marche, ITALY

## Abstract

Delivery is not easily predictable in horses and the consequences of dystocia can be serious for both the mare and foal. An induction protocol with low doses of oxytocin has been reported as a safe procedure. This study investigates the effect of induced delivery on at-term mares’ sympathetic-vagal balance. Fourteen mares were included and divided into two groups, one subjected to spontaneous delivery (SD), and one to induced delivery (ID). In both groups, an ECG was recorded using an elastic belt with integrated smart textile electrodes. The recording started before the delivery (Basal), continued close to delivery (Pre-delivery) and during delivery (Delivery), and ended after parturition (Placental expulsion). From the ECGs, Heart Rate Variability (HRV) parameters relating to time and frequency domains and non-linear analysis were extrapolated. The HRV analysis was performed both within the same group (IntraGA) and between the two groups (InterGA). In the present study, spontaneous and induced delivery did not appear to differ in autonomic nervous system functioning. In IntraGA analysis, both for SD and ID mares, delivery and placental expulsion periods were parasympathetic dominated since vagal-related HRV parameters increased. Moreover, no differences were found in InterGA comparison between SD and ID mares, except for the pre-delivery period of ID mares, during which both branches of the autonomic nervous system were activated. These results are in line with the literature on parasympathetic dominance during parturition and no change in Heart Rate Variability following exogenous oxytocin administration in parturient mares.

## 1. Introduction

In equine species, foaling is considered a critical event because the length of gestation is variable [1], and the signs of impending parturition are not clear and constant [2]. Moreover, the length of stage II of the delivery can be life-threatening for both the foal and mare in the case of dystocia [3].

In mares, induced labour is mainly performed in the presence of stud farm personnel and, in the case of high-risk pregnancies, in the presence of veterinary assistance. Most foaling happens during the night [4] when the possibility of assistance is lower. Induced foaling can thus be scheduled at a more practical time of the day.

Oxytocin (OX) is usually administered to induce labour in mares, using different dosages [5]. However, induced delivery with a low dose of oxytocin is considered, by many authors, as the safest approach both for mares and foals [2, 5, 6].

Oxytocin is physiologically released during delivery through a reflex mechanism due to the dilatation of the cervix and vagina caused by the foetus [1]. In rodents and humans, this hormone seems to have an anti-stress effect, with a downregulation of the sympathetic nervous system (SNS), and a restorative and protective function of the vagal system [7]. In humans, the exogenous administration of oxytocin has been shown to have an anxiolytic and sedative effect [8] with a shift towards the activation of the parasympathetic nervous system (PNS) [9] and an increase in heart rate variability (HRV). In women [9, 10], administration of exogenous OX to induce labour can lead to a dose-dependent bradycardic effect and an increase in HRV spectral power, as found by Weissman and colleagues in a clinical setting [10].

The HRV index represents the short fluctuations of HR and is used to estimate the influence of the sympathetic or vagal branch on the sinus node. HRV analysis is performed by identifying R-R peaks on an ECG recording [11], and HRV parameters differ in the time-domain, frequency-domain, and non-linear categories. The parameter values for each category can be used to indicate whether the organism has a greater SNS or PNS shift [12]. An increase in the HRV parameters is considered representative of vagal activation. On the other hand, a decrease in the HRV parameters may reflect a shift in the activation of the autonomic nervous system (ANS) toward the sympathetic branch [11, 13–15].

It is thus very important to have a good quality ECG recording. This can be achieved using smart textile electrodes, which are comfortable and easy to use [16], provide high-quality signals, reduce ECG artefacts, and simplify and improve data analysis [17].

The present study aimed to compare the HRV parameters assessed in mares during spontaneous and induced parturition with a low dose of oxytocin. The hypothesis was that the induction of foaling might influence the sympathetic-vagal balance.

## 2. Materials and methods

### 2.1. Animals and management

The study was conducted on 14 trotter mares, aged between 7–18 years (median age: 11.5 years) from a stud farm associated with the Veterinary Teaching Hospital of the Department of Veterinary Sciences (University of Pisa, Italy), during one foaling season. All the mares enrolled in this study were housed in the stud farm because the owners requested attended parturition.

The inclusion criteria for the mares were: 1) healthy based on a general physical examination performed during the last week of gestation; 2) foetal anterior presentation confirmed by ultrasound examination at admission; 3) multiparous pregnancies; 4) similar management.

Mares were divided into two groups: spontaneous delivery (SD) and induced delivery (ID). Each group was composed of seven mares, all bedded on straw and fed with hay *ad libitum* and flaked grain. They were checked daily, turned out in the paddock during the day, and moved to a foaling box during the night for continuous monitoring with video cameras as soon as udder enlargement was detected and/or when day 320 of gestation was reached. A commercial foaling alert system (C6 Birth Control, Sisteck, Italy) was attached to the vulva, one week before the estimated date of foaling, and connected to the breeder’s phone. The calcium concentration in mammary secretion was evaluated at 5:00 pm each day, starting when the mammary secretion changed from watery to sticky, using a Foal Watch^®^ Test Kit (Chemetrics, USA).

In the ID group, the delivery was induced as previously described [2] with a single injection of 2.5 IU of oxytocin (Neurofisin^®^, Fatro, Italy) IV at 18:15 when calcium concentrations reached ≥ 250 ppm [18]. The mares showing no signs of labour within 60 min of treatment were judged not to be ready to foal and were not injected again. Treatment was repeated once a day until delivery.

The expulsion of the foetus was assisted, in both spontaneous and induced foaling, with gentle traction by an operator on the foetus’ front limbs.

Information on parity, gestational length, length of stage II of parturition (considered as the interval between the rupture of the allantoic sac and the complete expulsion of the foal) and placental expulsion were recorded. Placenta retention was also assessed, defined as no expulsion of the foetal membrane within three hours of foaling [19, 20].

Foal viability was evaluated using a 5-parameter Apgar score [21] five minutes after birth. In addition, heart rate [22] (normal >60 beats/min), respiratory rate [22] (normal 60–80 breaths/min), and rectal body temperature (BT) [23] (normal 37.2–38.9°C) values were recorded. Foal maturity was assessed as previously reported [24]. Behavioural parameters were also recorded, such as time to acquire sternal recumbency (normal <2 minutes), standing position (normal <120 minutes) and nursing from dams (normal <240 minutes) [22]. The foals were clinically evaluated at least twice a day for up to seven days of life.

### 2.2. Electrocardiogram recording

Electrocardiograms (ECGs) were recorded using an elastic belt with integrated textile electrodes (Smartex SRL, Pisa, Italy). The belt was placed behind the wither using two straps, one at the sternum, and the other at the caudal abdomen. The belt had a small pocket in which the recording device (Smartex SRL, Pisa, Italy) was placed. The textile electrodes (13x5 cm) were positioned in a modified base-apex configuration. One electrode was placed behind the shoulder blades in the dorsal half-height, while the other was positioned about 20 cm behind and 10 cm above the elbow.

**Fig 1:**
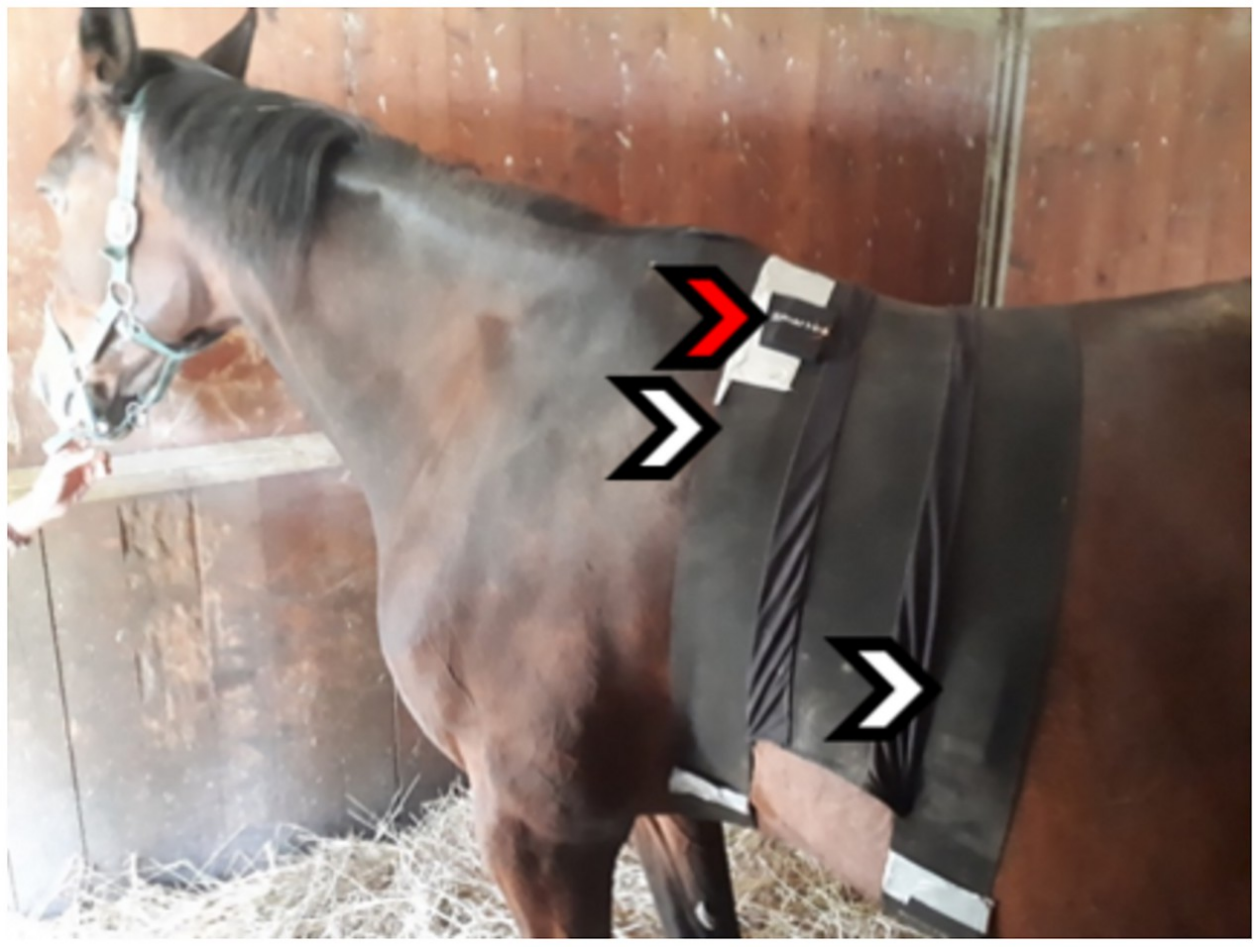
Position of the belt with integrated textile electrodes (Smartex SRL, Pisa, Italy). Red Arrow electronic system and White Arrow textile electrodes.

When fitting the belt, the straps were coupled and attached with sellotape (Fig 1). The electrodes were then wetted with water and the recording system was turned on and connected through wires to the textile electrodes.

The signal was sent via Bluetooth to a portable tablet and checked in real-time every five minutes.

The ECGs were recorded using the following protocols:

- SD group: recording started at 8:00 pm and stopped at 6:00 am if the delivery did not occur, or at the moment of placenta expulsion in the case of foaling.
- ID group: recording started at 6:00 pm and stopped at the moment of placenta expulsion, after foaling. The oxytocin was injected 15 min after the beginning of the recording.

Four different periods [25] were recognised within the ECGs:

1. Basal: the period started with the beginning of the ECG recording for both groups (SD and ID) and ended 1h before the rupture of the chorioallantoic membrane (lower limit for the onset of stage I of parturition) for SD mares and at the time of oxytocin injection for the ID mares. During this period the mare was left alone in the box.
2. Pre-delivery: the period started 1h before the rupture of the chorioallantoic membrane for the SD mares or at the time of oxytocin injection for the ID mares and ended with the rupture of the chorioallantoic membrane for both groups. This represents the time just before delivery (stage I of parturition). During this period the mare was left alone in the box.
3. Delivery: the period started with the rupture of the chorioallantoic membrane and ended with the expulsion of the foal (stage II of parturition) in both groups. During this period one operator assisted the foetus’s expulsion by gentle traction of the foetus’s forelimbs.
4. Placental expulsion: the period started after the completion of foetal expulsion and ended with the placental expulsion (stage III of parturition). During this period, both the mare and foal were left alone in the box.

Within these four periods, 4 windows were selected (one for each period), all lasting 5 minutes (both for SD and ID groups). From each of the four windows, HRV parameters were extracted. The four windows were collected, respectively:

- at the farthest end from the delivery event, in the basal period.
- in the middle of the pre-delivery period.
- at the beginning of the delivery period, including the actual delivery event.
- at the end of the placental expulsion period, including the actual placental expulsion event.

### 2.3. Data analysis

Data concerning gestational and stage II labour were reported as median, minimum, and maximum values. ECG signals were processed by Matlab software (MATLAB Environment, 2021) to extract the HRV time series. Since the computation of some ECG extracted parameters is dependent on the time-window length, the ECG signal was analysed within time windows of equal length for all the animals to obtain comparable results in each period. All ECGs were also pre-processed using a bandpass filter to remove noise and movement artefacts. From QRS complexes, R-peaks were detected and then used to obtain the InterBeat Interval (IBI), that is the time interval between individual beats, whose variations over time are known as Heart Rate Variability.

The HRVs were analysed in the time domain, frequency domain, and by a non-linear method. In the time domain, from heart rate (HR) and HRV, we extracted meanRR, SDNN and RMSSD parameters. In the frequency domain, the power spectrum was computed in three specific frequency bandwidths, namely LF, HF and LF/HF. As a non-linear HRV analysis, Poincaré plot analysis was performed; in particular, the SD1 and SD2, (the two well-known Poincaré plot parameters) were computed [26]. Quantitative analysis of the plot shape was performed [27] using R-R segments extrapolated from each of the four windows analysed. The description of the parameters analysed, and their physiological meaning were reported in Table 1.

**Table 1:** Name, unit, description, and physiological meaning of the HR and HRV parameters included in the present study (modified from Shaffer et al, [12]).

*Parameter name*	*Unit*	*Description*	*Physiological meaning*
MeanRR	ms	Mean of R-R intervals	A longer meanRR interval means a lower heart rate
SDNN	ms	Standard deviation of R-R intervals	Represents both cardiac sympathetic and parasympathetic activity
RMSSD	ms	Root mean square of successive R-R differences	Used to estimate the vagally-mediated changes reflected in HRV. A high RMSSD value means a higher cardiac parasympathetic activity
LF	ms2	Absolute power of the low-frequency band (0.07–0.6 Hz)	Represents both cardiac sympathetic and parasympathetic activity
HF	ms2	Absolute power of the high-frequency band (0.01–0.07 Hz)	Reflects mainly cardiac parasympathetic activity. A high HF value means a higher parasympathetic cardiac activity
LF/HF	%	Ratio of LF-to-HF power	A low LF/HF ratio reflects cardiac parasympathetic activity, and a high LF/HF ratio indicates cardiac sympathetic activity
SD1	ms	Poincaré plot standard deviation perpendicular to the line of identity	Is known to be linked to the time domain parameter RMSSD. It mainly represents the cardiac parasympathetic activity
SD2	ms	Poincaré plot standard deviation along the line of identity	Is known to be linked to the time domain parameter SDNN. It represents both cardiac sympathetic and parasympathetic activity

ms: millilseconds; ms^2^: milliseconds squared divided by cycles per second; %: percentage of the total HRV power.

The HRV normality distribution was assessed through the Shapiro-Wilk test in each window within the four periods (basal, pre-delivery, delivery, and placental expulsion). Since the distribution of the feature samples was not Gaussian, nonparametric statistical tests were adopted.

Two statistical analyses were performed: an intra-group analysis (IntraGA) (i.e., within SD and ID groups, respectively) and an inter-group analysis (InterGA) (comparing the two groups for each protocol window within the four periods).

For the IntraGA, the Friedman test and the Bonferroni-corrected Wilcoxon signed-rank test as post-hoc were applied on HRV parameters to verify significant differences in each pairwise comparison among the four different periods analysed (basal vs. pre-delivery vs. delivery vs. placental expulsion).

For the InterGA, a Bonferroni-corrected Mann-Whitney test for unpaired data was used to assess statistical differences between ID and SD groups for each period analysed (basal, pre-delivery, delivery, and placental expulsion).

The significant value was set for p<0.05. The statistical analysis was performed using commercial software (MATLAB Environment, 2021).

### 2.4. Ethical note

The research protocol was approved by the Institutional Animal Care and Use Committee of the University of Pisa (33476/2016). Owner-informed consent was obtained for each horse.

## 3. Results

The median gestational length was within the reference interval (320–360 days) [28] with a median value of 340 and 335 days for the SD and ID groups, respectively. A single dose of OX was sufficient to induce parturition in all the mares in the ID group. In the ID mares, the expulsion of the foetus was completed in a median time of 62 minutes (43–99 minutes) after the OX injection. The median length of the stage two parturition was 9 minutes in both groups; the minimum and maximum values were 6–29 minutes and 4–21 minutes in the SD and ID groups, respectively. No statistical differences (p = 0.875) were observed between the two groups.

No dystocia occurred in the SD Group, while one premature separation of the placenta (red bag) was recorded in the ID group; the chorioallantoic membrane was immediately broken and the foetus was safely delivered. Foetal membranes were retained over 3 hours and in one mare in both groups, spontaneously expelled within 12 hours.

Foals of both groups were born alive and showed no signs of prematurity/dysmaturity. Four out of 7 fillies and 3/7 colts were born after spontaneous delivery and 5/7 fillies and 2/7 colts after induced delivery. All the foals were clinically healthy based on a daily physical examination performed up to 7 days of age. In the SD group, the Apgar score was 9–10 in all the foals; in the ID group, the Apgar score was 9–10 in 6/7 foals, while 1/7 showed an Apgar score of 8. No statistical differences were found in viability between the two groups (p = 0.192).

In the SD group, more time was needed to acquire the sternal recumbency and the standing position in 2/7 and 1/7 foals, respectively, while no foals showed a prolonged time until the first suckling. In the ID group, a prolonged time for sternal recumbency and acquiring a standing position was found in 3/7 foals, respectively, while in 1/7 foals, the time until the first suckling was prolonged. In the SD group all the foals showed HR, RR and BT values within the reference ranges, while in the ID group, 3/7 foals showed a lower RR. Statistical differences were not found for time to sternal recumbency (p = 0.2552), time to acquire standing position (p = 0.0775) and to suckle (p = 0.2576), for either HR (p = 0.8322) or RR (p = 0.1498). Statistical differences were found in BT values (37.6 vs 38.1 in the SD vs ID foals, respectively) (p = 0.0006).

Regarding HRV analysis, the extracted HRV parameters were expressed as median values and absolute deviation from the median (MAD), both for SD and ID groups, for each period analysed and reported in Table 2.

**Table 2:** HRV features obtained for each analyzed period and for both groups. Data are expressed as median and absolute deviation from the median. Within the same row, different superscripts mean statistical differences over time (intraGA): a ≠ b ≠ c ≠ d (all p < 0.008). The absence of superscripts within the same row means no statistical differences over time.

**SD GROUP**				
**HRV feature**	**Basal**	**Pre-Delivery**	**Delivery**	**Placental expulsion**
Mean RR	1.056^a^	1.028^a^	1.229^ab^	1.351^b^
ms	[0.069]	[0.111]	[0.124]	[0.106]
SDNN	0.093^a^	0.084^ab^	0.311^c^	0.353^ac^
ms	[0.021]	[0.027]	[0.685]	[0.308]
RMSSD	0.039^a^	0.051^a^	0.327^b^	0.480^ab^
ms	[0.041]	[0.056]	[0.950]	[0.405]
LF	0.001^a^	0.002^ab^	0.021^c^	0.016^b^
ms^2^	[0.000]	[0.001]	[5.949]	[0.451]
HF	0.000^a^	0.001^ab^	0.021^c^	0.047^b^
ms^2^	[0.001]	[0.002]	[0.548]	[0.081]
LF/HF	2.641	2.814	0.719	0.923
%	[4.136]	[1.505]	[2.455]	[1.449]
SD1	0.028^a^	0.037^a^	0.231^b^	0.339^ab^
ms	[0.029]	[0.039]	[0.069]	[0.286]
SD2	0.107^a^	0.114^a^	0.374^b^	0.365^b^
ms	[0.025]	[0.022]	[0.676]	[0.334]
**ID GROUP**				
**HRV feature**	**Basal**	**Pre-Delivery**	**Delivery**	**Placental expulsion**
Mean RR	0.944^a^	0.867^b^	1.014^ab^	1.119^c^
ms	[0.089]	[0.095]	[0.114]	[0.149]
SDNN	0.076^a^	0.058^a^	0.123^b^	0.129^ab^
ms	[0.017]	[0.011]	[0.083]	[0.054]
RMSSD	0.077^ab^	0.044^a^	0.143^b^	0.076^b^
ms	[0.020]	[0.026]	[0.134]	[0.091]
LF	0.001^ab^	0.001^a^	0.003^b^	0.003^ab^
ms^2^	[0.001]	[0.000]	[0.008]	[0.002]
HF	0.001^ab^	0.000^a^	0.003^b^	0.001^ab^
ms^2^	[0.001]	[0.001]	[0.012]	[0.006]
LF/HF	0.951	2.078	0.821	1.734
%	[0.378]	[1.165]	[0.359]	[0.629]
SD1	0.054^ab^	0.031^a^	0.101^b^	0.053^b^
ms	[0.014]	[0.018]	[0.094]	[0.067]
SD2	0.096^a^	0.071^a^	0.141^b^	0.176^ab^
ms	[0.021]	[0.009]	[0.078]	[0.054]

ms: millilseconds; ms^2^: milliseconds squared divided by cycles per second; %: percentage of the total HRV power.

The IntraGA analysis showed statistical differences in SD group for basal and pre-delivery periods vs. delivery and placental expulsion periods. In particular, SDNN, RMSSD, LF, HF, SD1, and SD2 values increased in the delivery vs. the pre-delivery and the basal period. Moreover, meanRR, LF, HF, and SD2 values increased in the placental expulsion vs. basal period, while meanRR, SDNN, and SD2 values raised in the placental expulsion vs. the pre-delivery period. All the statistical significance had a p-value of 0.008.

For the ID group, the IntraGA analysis showed a statistically significant reduction in meanRR value in pre-delivery period, an increase in SDNN and SD2 values in delivery period, and an increase in meanRR value in placental expulsion period, all compared to basal. In addition, an increase in SDNN, RMSSD, LF, HF, SD1, and SD2 values for delivery vs. pre-delivery, and an increase in meanRR, RMSSD, and SD1 values for placental expulsion vs. pre-delivery was found. Lastly, meanRR value increased in the placental expulsion period vs. the delivery period. All the statistical significance had a p-value of 0.008.

The InterGA statistical analysis showed a significant decrease in SDNN (p = 0.009), LF (p = 0.004) and SD2 (p = 0.004) values in pre-delivery period of the ID vs. SD group.

## 4. Discussion

Oxytocin is the drug of choice for induced parturition in mares and is widely considered as safe and easy-to-use when employed at a dosage of 2.5/3.5 UI [2, 6, 29].

In horses, induced parturition, by PGF2 alpha or oxytocin at high or repeated doses until foaling can be related to complications, such as dystocia, premature placental separation, retained placental membranes, foetal hypoxia, or failure of passive transfer [5, 30–33]. The number of mares included in our study was too low to evaluate the prevalence of such complications. Although a case of premature placental separation (ID group) and two cases of retained foetal membranes (one in each group) were observed, in a previous study no statistical differences in the II stage length nor in incidence of peripartum complications were observed between spontaneous vs induced parturition [34].

In horses, the main problem related to induced delivery is the increased risk of foal prematurity [5]. In our study, all induced mares had exceeded 330 days of pregnancy, and the pre-induction maturity of the foetus was determined by evaluating the calcium content in the mammary secretion. Newborn foals were assessed for maturity using the criteria suggested by Rossdale and colleagues [24] showing no differences in viability (APGAR score), physical and behavioural parameters after spontaneous or induced parturition except for body temperature which was lower in the spontaneous delivery group. These observations are in line with other studies on inducing foaling using low doses of oxytocin, which reported no major complications either for the mares or the newborn foals [2, 6, 29, 35].

In this study, we hypothesized that the treatment with exogenous oxytocin to induce foaling in at-term mares might influence their sympathetic-vagal balance. To the best of our knowledge, no studies evaluated the HRV after the administration of oxytocin to induce parturition in mares.

Overall, in the SD group IntraGA comparison, cardiac parasympathetic activity seems to be more represented in delivery and placental expulsion periods compared to basal and pre-delivery ones (see Table 2). This is in line with the literature, especially regarding delivery, which is known to be dominated by parasympathetic activity in horses [36]. The parasympathetic activation we found in the placental expulsion period appears to be partially in line with other studies reporting that parasympathetic tone during the third stage was still high, although decreasing compared to the second stage [36–38]. In this study, HRV vagal parameters recorded during the placental expulsion period were not statistically different from those found during the delivery period. This could be because the window of the placental expulsion period in which HRV was analysed included the actual event of foetal membrane release. Our hypothesis is that foetal membrane release may have induced a spike in oxytocin production and thus parasympathetic cardiac activation equal to that of delivery [39].

Moreover, in SD group the influence of ANS on cardiac function seems to be the same in basal vs pre-delivery periods. This is not surprising, as parasympathetic activity starts increasing 30 minutes before delivery and then peaks 15 minutes after [36]. The lack of statistically significant differences between basal and pre-delivery HRV values may be due to a minimal, not yet marked increase in the pre-delivery HRV values (see Table 2).

In IntraGA comparison, for ID group, the HRV increased from basal and pre-delivery to delivery and placental expulsion periods (see Table 2). This agrees with what we found for SD group mares. However, in ID group the HRV decreased from basal to pre-delivery period (see Table 2). This is not in line with the literature [39, 40], nor with what we found for SDs. Usually, oxytocin favours parasympathetic function [39, 40], and in our case, the administration of oxytocin for induction of delivery should have resulted in increased HRV values. The decrease in HRV values found in this study following oxytocin injection could be due to manipulation-related sympathetic activation or the injection itself, which could have triggered a fight-or-flight response [36]. In fact, studies investigating different types of manipulations, namely transrectal palpation and administration of drugs in different formulations, found that these manipulations had an influence on the sympathetic-vagal balance, stimulating increased sympathetic activation [41, 42]. Further investigations would be necessary to assess the possible effect of injections and related manipulations on the ANS. Finally, the increase in HRV values in the placenta expulsion vs. parturition period for ID mares could be related to the fetal membranes release event, as discussed previously.

When the two groups were compared (IntraGa comparison), no differences were found between periods, except for the pre-delivery one. In particular, in ID mares HRV tended to decrease during the pre-delivery period in those parameters representative of both sympathetic and parasympathetic activity (see Table 2). Coactivation of both branches of the ANS is known as a mitigated stress response. When an animal is exposed to a stressful event, an initial context validation occurs, followed by an increased sympathetic activation (full stress response), or an increased parasympathetic activation (relaxation). How the initial coactivation evolves depends on whether the animal can predict and control the stressor [41]. In our study, the mares were all adults and multiparous. It is therefore likely that they already experienced manipulations related to drug administration. This may have induced the coactivation of the two branches of the ANS. However, further parameters, including behaviour, would be necessary to confirm the possible impact of the manipulations on the sympathetic-vagal balance.

In our study, spontaneous and oxytocin-induced delivery did not appear to differ in terms of autonomic nervous system functioning and did not alter the physiological course of the events of delivery. This is in line with what has been found in other studies [39, 40]. The only difference found between spontaneous and induced delivery was in the pre-delivery period of ID mares. This could be due to manipulations and injection of the drug since the trend of the ANS was diametrically opposite to what was expected following oxytocin administration [40]. Moreover, as shown by numerous other studies [15, 36, 39], we found that delivery in the equine is a parasympathetic-dominated event. However, unlike other studies, in the postpartum, we found an increase in the parasympathetic domain, which seemed to be related to the fetal membrane expulsion event. Although oxytocin release related to placental expulsion is known in human medicine [43], this aspect should be investigated further in the equine species.

Our study certainly has limitations, which consist of the low number of subjects monitored and the electrocardiographic monitoring performed so close to delivery. Undoubtedly, increasing the number of subjects involved and the hours of electrocardiographic recording both before and after delivery may be useful implementations to be applied in further studies on the evolution of autonomic nervous system functioning during spontaneous and induced delivery in the equine species.

## 5. Conclusions

We confirmed that induced parturition with low doses of oxytocin is safe both for mares and foals, in line with several other reports [2, 6, 29, 35, 39].

The hypothesis that the treatment with exogenous oxytocin to induce foaling in at-term mares might influence their sympathetic-vagal balance was not fully confirmed. In our study, we found no differences in mares’ autonomic nervous system function during spontaneous or induced delivery. The only difference found was a decrease in HRV in the pre-delivery of mares whose foaling was induced, and this could be attributable to the manipulations the mares were subjected to during drug administration. Parturition and placental expulsion appear to be dominated by parasympathetic functioning in both induced and spontaneous delivery mares. In conclusion, our findings suggest that the physiology of the parturition was preserved by applying a delivery induction protocol with a low dose of oxytocin.

## Supporting information

S1 File (XLSX)
